# Type III interferons disrupt the lung epithelial barrier upon viral recognition

**DOI:** 10.1126/science.abc3545

**Published:** 2020-06-11

**Authors:** Achille Broggi, Sreya Ghosh, Benedetta Sposito, Roberto Spreafico, Fabio Balzarini, Antonino Lo Cascio, Nicola Clementi, Maria De Santis, Nicasio Mancini, Francesca Granucci, Ivan Zanoni

**Affiliations:** 1 Harvard Medical School, Boston Children’s Hospital, Division of Immunology, Boston, MA, USA.; 2 Department of Biotechnology and Biosciences, University of Milano-Bicocca, Milan, Italy.; 3 Institute for Quantitative and Computational Biosciences, University of California, Los Angeles, CA, USA.; 4 Laboratory of Medical Microbiology and Virology, Vita-Salute San Raffaele University, Milan, Italy.; 5 Department of Rheumatology and Clinical Immunology, Humanitas Clinical and Research Center - IRCCS, Rozzano, Italy.; 6 IRCCS San Raffaele Hospital, Milan, Italy.; 7 National Institute of Molecular Genetics (INGM) “Romeo ed Enrica Invernizzi,” Milan, Italy.; 8 Harvard Medical School, Boston Children’s Hospital, Division of Gastroenterology, Boston, MA, USA.

## Abstract

Viral infections of the lower respiratory tract are a leading cause of mortality. Mounting evidence indicates that most severe cases are characterized by aberrant immune responses and do not depend on viral burden. In this study, we assessed how type III interferons (IFN-λ) contribute to the pathogenesis induced by RNA viruses. We report that IFN-λ is present in the lower, but not upper, airways of patients with coronavirus disease 2019 (COVID-19). In mice, we demonstrate that IFN-λ produced by lung dendritic cells in response to a synthetic viral RNA induces barrier damage, causing susceptibility to lethal bacterial superinfections. These findings provide a strong rationale for rethinking the pathophysiological role of IFN-λ and its possible use in clinical practice against endemic viruses, such as influenza virus as well as the emerging severe acute respiratory syndrome coronavirus 2 (SARS-CoV-2) infection.

## Interferons interfere with lung repair

Interferons (IFNs) are central to antiviral immunity. Viral recognition elicits IFN production, which in turn triggers the transcription of IFN-stimulated genes (ISGs), which engage in various antiviral functions. Type I IFNs (IFN-α and IFN-β) are widely expressed and can result in immunopathology during viral infections. By contrast, type III IFN (IFN-λ) responses are primarily restricted to mucosal surfaces and are thought to confer antiviral protection without driving damaging proinflammatory responses. Accordingly, IFN-λ has been proposed as a therapeutic in coronavirus disease 2019 (COVID-19) and other such viral respiratory diseases (see the Perspective by Grajales-Reyes and Colonna). Broggi *et al.* report that COVID-19 patient morbidity correlates with the high expression of type I and III IFNs in the lung. Furthermore, IFN-λ secreted by dendritic cells in the lungs of mice exposed to synthetic viral RNA causes damage to the lung epithelium, which increases susceptibility to lethal bacterial superinfections. Similarly, using a mouse model of influenza infection, Major *et al.* found that IFN signaling (especially IFN-λ) hampers lung repair by inducing p53 and inhibiting epithelial proliferation and differentiation. Complicating this picture, Hadjadj *et al.* observed that peripheral blood immune cells from severe and critical COVID-19 patients have diminished type I IFN and enhanced proinflammatory interleukin-6– and tumor necrosis factor-α–fueled responses. This suggests that in contrast to local production, systemic production of IFNs may be beneficial. The results of this trio of studies suggest that the location, timing, and duration of IFN exposure are critical parameters underlying the success or failure of therapeutics for viral respiratory infections.

*Science*, this issue p. 706, p. 712, p. 718; see also p. 626

## Teaser

Interferons can both enhance and abate the severity of respiratory viral infections.

The ability to resolve viral infections of the lung is dependent on the actions of interferons (IFNs) and inflammatory cytokines, yet their relative contributions to host defense and return to homeostasis remain undefined. In particular, type III IFNs (IFN-λ) have attracted much attention, as they operate primarily at mucosal surfaces (*1*). Recent work established that, unlike other IFNs, IFN-λ signaling induces antiviral activities while simultaneously limiting the tissue-damaging functions of neutrophils (*2*–*4*). When considered in the context of respiratory viral infections in which inflammation appears to be the primary driver of life-threatening symptoms, including the recently emerged severe acute respiratory syndrome coronavirus 2 (SARS-CoV-2) (*5*), the ability of IFN-λ to limit immunopathology but maintain antiviral activity is noteworthy. Discussions on the possible use of IFN-λ against SARS-CoV-2 have begun (*6*), and clinical trials have been initiated. However, despite this interest in the use of IFN-λ to treat viral infections, the long-term effects of IFN-λ on lung physiology remain largely overlooked. For example, during viral infections of the lung, immunopathology may predispose the host to opportunistic bacterial infections, and IFN-λ impairs bacterial control during superinfections (*7*, *8*). It remains unresolved whether this is due to the anti-inflammatory activity of IFN-λ, which reduces host resistance, or to the capacity of IFN-λ to alter lung physiology upon a viral encounter. Indeed, superinfections represent the first cause of lethality upon influenza virus infection (*9*) and correlate with severity in coronavirus disease 2019 (COVID-19) patients (*10*).

**Fig. 1:**
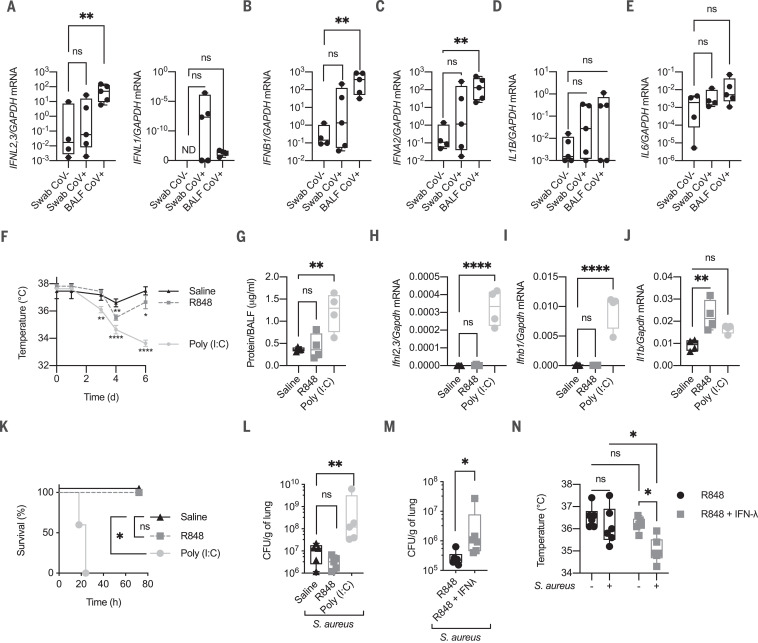
Morbidity correlates with the high expression of type I IFN and IFN-λ in the lung of COVID-19 patient BALF and of poly (I:C)–treated mice. (**A** to **E**) *IFNL2,3*, *IFNL1* (A), *IFNB* (B), *IFNA2* (C), *IL1B* (D), and *IL6* (E) mRNA expression was evaluated in naso-oropharyngeal swabs from SARS-CoV-2–positive (Swab CoV^+^) and –negative (Swab CoV^−^) participants and from the BALF of intensive care unit (ICU)–hospitalized SARS-CoV-2–positive patients (BALF CoV^+^) (five participants per group). GAPDH, glyceraldehyde phosphate dehydrogenase; ND, not detectable. (**F** to **J**) Mice were intratracheally (i.t.) administered 2.5 mg of poly (I:C) per kilogram of body weight, 2.5 mg of R848 per kilogram of body weight, or saline daily for 6 days. (F) Body temperatures of the treated mice measured over time. (G) Amount of total protein in the BALF measured after 6 days of poly (I:C) treatment. (H to J) *Ifnl2,3* (H), *Ifnb1* (I), and *Il1b* (J) mRNA expression was assessed in total lung lysate harvested 6 days after treatment. (**K** and **L**) Mice treated as in (F) to (J) were infected at day 6 with 5 × 10^7^ colony-forming units (CFU) of *S. aureus* administered i.t. and were monitored for survival (K). Bacterial loads in the lungs of the treated mice normalized to lung weight were assessed 12 hours postinfection (hpi) (L). Mice were i.t. administered R848 (2.5 mg/kg) or a combination of R848 and IFN-λ (50 μg/kg) daily for 6 days and were then infected as in (K). Lung bacterial burdens (**M**) and body temperatures (**N**) before and after *S. aureus* infection are shown [(G) to (J), (L) to (N)]. Each symbol represents one mouse. The median and range are represented. (F) Means ± SDs of five mice per group are represented. (G to J) Four, (L and M) five, and (N) six mice per group are represented, and median and range are shown. (K) Survival plot of five mice per group. (F to N) Representative data of three independent experiments. Statistics: ns, not significant (*P* > 0.05); *P < 0.05; **P < 0.01; ****P < 0.0001. Two-way analysis of variance (ANOVA) [(F) and (N)], one-way ANOVA [(G) to (J), (L)], or two-tailed *t* test (M) was performed. Logarithmic values were fitted when evaluating bacterial load [(L) and (M)]. Log-rank (Mantel-Cox) test, corrected for multiple comparisons, was performed to evaluate survival (K).

Mouse models of SARS, Middle East respiratory syndrome (MERS) (*11*, *12*), and influenza (*1*, *13*) are characterized by a robust induction of type I and III IFNs. However, the involvement of these cytokines in COVID-19 is controversial (*14*, *15*). To directly evaluate the capacity of SARS-CoV-2 to induce IFNs, we tested naso-oropharyngeal swabs of COVID-19 patients and healthy controls, as well as the bronchoalveolar lavage fluid (BALF) of SARS-CoV-2–positive patients with severe COVID-19. Levels of IFN mRNAs in the upper airways of COVID-19 patients were not significantly different from levels in healthy controls. By contrast, BALF of patients with severe disease presented elevated levels of both inflammatory cytokines as well as type I and III IFNs (Fig. 1, A to E).

To evaluate the contribution of IFN-λ to the immunopathology driven by RNA respiratory viruses uncoupled from its effect on viral replication, we devised an experimental system in which pattern recognition receptors (PRRs) involved in viral sensing were stimulated with their cognate ligands. RNA viruses are sensed via either endosomal Toll-like receptor (TLR) 3 and TLR7 or cytoplasmic retinoic acid–inducible gene I (RIG-I) and melanoma differentiation-associated protein 5 (MDA5) (*16*). We intratracheally instilled the TLR7 ligand, R848, or the synthetic analog of double-stranded RNA, polyinosine:polycytidylic acid [poly (I:C)], that stimulates both TLR3 and the RIG-I–MDA5 pathway in vivo (*17*). PRRs were stimulated over the course of 6 days to elicit prolonged innate immune activation in the lung. Both ligands induced hypothermia (Fig. 1F) and weight loss (fig. S1A), but only poly (I:C) compromised barrier function (Fig. 1G and fig. S1B). IFN mRNAs were strongly up-regulated by poly (I:C) but not R848 (Fig. 1, H and I). By contrast, R848 treatment induced the up-regulation of proinflammatory cytokines (i.e., *Il1b*), but this did not correlate with barrier function decrease (Fig. 1, G to J, and fig. S1B).

Alterations in the epithelial barrier predispose mice to lethal bacterial superinfections (*18*). We therefore infected mice treated with either R848 or poly (I:C) with *Staphylococcus aureus*. Mice treated with poly (I:C) died upon *S. aureus* infection (Fig. 1K) and had higher bacterial burdens (Fig. 1L), more intense hypothermia, and greater barrier damage (fig. S2, A and B). *S. aureus* infection did not alter the pattern of cytokine expression compared to that in mice treated with viral ligands only (fig. S2, C to E). Upon poly (I:C) administration, IFN-β and IFN-λ transcript and protein levels were rapidly up-regulated and plateaued (fig. S3, A to D), whereas *S. aureus* bacterial burden increased with consecutive injections of poly (I:C) (fig. S3E). IFN-stimulated genes, but not proinflammatory cytokines, were also sustained over time (fig. S3, F to I). These data suggest that chronic exposure to IFNs aggravates bacterial superinfections. Because the protein levels of IFN-λ were very high compared with those of IFN-β (fig. S3, C and D), we assessed whether IFN-λ was sufficient to exacerbate bacterial superinfections. We administered exogenous IFN-λ either alone, or with R848, which induces inflammation but not IFN production (Fig. 1, H to J). The administration of IFN-λ with R848, but not IFN-λ alone, was sufficient to induce sensitivity to *S. aureus* infection (Fig. 1, M and N, and fig. S3J). Thus, in an inflamed lung, IFN-λ is sufficient to aggravate superinfections.

**Fig. 2:**
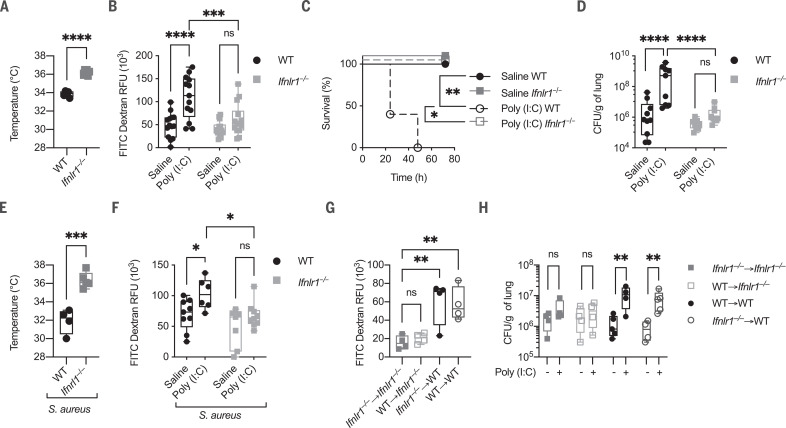
IFN-λ is necessary to increase susceptibility to bacterial infection induced by antiviral immunity. (**A** and **B**) WT and *Ifnlr1^−/−^* mice were i.t. treated with 2.5 mg/kg poly (I:C) or saline daily for 6 days. (A) Body temperatures of poly (I:C)–treated WT and *Ifnlr1^−/−^* mice were recorded on day 6. (B) On day 6, mice were i.t. treated with fluorescein isothiocyanate (FITC)–dextran (10 μg per mouse). Barrier permeability was measured as relative fluorescent units (RFU) of FITC-dextran leaked in plasma 1 hour after injection. (**C** to **F**) WT and *Ifnlr1^−/−^* mice i.t. treated with 2.5 mg/kg poly (I:C) or saline for 6 days were i.t. infected with 5 × 10^7^ CFU of *S. aureus* and monitored for survival (C). Lung bacterial burdens normalized by lung weight (D), body temperature (E), and barrier permeability (F) [as in (B)] were assessed 12 hpi. (**G** and **H**) Lethally irradiated WT or *Ifnlr1^−/−^* recipients were reconstituted with donor bone marrow (*Ifnlr1^−/−^* or WT) for 6 weeks and were then treated as in (C) to (F). Resulting chimeric mice were defective for IFN-λ signaling in either the hematopoietic compartment (*Ifnlr1^−/−^* → WT) or in the stromal compartment (WT → *Ifnlr1^−/−^*). *Ifnlr1^−/−^* → *Ifnlr1^−/−^* and WT → WT chimeras were used as controls. (G) Barrier permeability [as in (B)] and (H) lung bacterial burdens were evaluated 12 hpi. Each symbol represents one mouse. The median and range are represented. (C) Survival plot of five mice per group. (A to H) Representative data of three independent experiments. (A, E, G, and H) Four, (B) 14, and (D and F) 10 mice per group; median and range are represented. Statistics: ns, not significant (*P* > 0.05); *P < 0.05; **P < 0.01; ***P < 0.001; ****P < 0.0001. Two-way ANOVA [(B), (D), (F), (H)], one-way ANOVA (G), or two-tailed *t* test [(A) and (E)] was performed. Logarithmic values were fitted when evaluating bacterial load [(D) and (H)]. Log-rank (Mantel-Cox) test, corrected for multiple comparisons, was performed to evaluate survival (C).

In contrast to wild-type (WT) mice, mice deficient in IFN-λ receptor 1 (*Ifnlr1*) expression were protected from poly (I:C)–induced morbidity and barrier damage (Fig. 2, A and B, and fig. S4, A and B). *Ifnlr1^−^*^/^*^−^* mice were also resistant to superinfection with *S. aureus* (Fig. 2, C to F). By contrast, the absence of *Ifnlr1* did not affect mRNA or protein levels of IFNs or proinflammatory cytokines (fig. S4, C to H). We next generated reciprocal bone marrow chimeras in which either the hematopoietic or the stromal compartments were defective for IFN-λ signaling. Absence of *Ifnlr1* in the stromal compartment, but not in hematopoietic cells, phenocopied complete *Ifnlr1* deficiency (Fig. 2, G and H, and fig. S5). Furthermore, there was no difference in myeloid immune cell recruitment in *Ifnlr1^−^*^/^*^−^* compared to WT mice (fig. S6, A to D), and depletion of neutrophils did not affect bacterial burden (fig. S6E). Thus, IFN-λ signaling in epithelial cells is necessary and sufficient to induce susceptibility to a secondary infection.

**Fig. 3:**
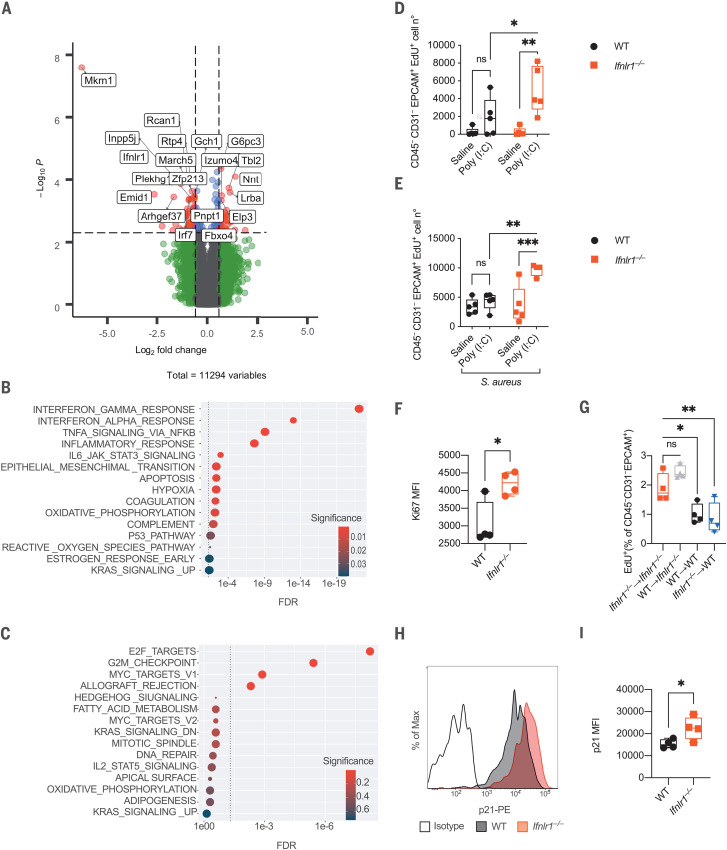
IFN-λ signaling directly inhibits lung epithelia proliferation and impairs repair upon viral recognition. (**A** to **C**) Targeted transcriptome sequencing was performed on lung epithelial cells isolated on day 6 from WT and *Ifnlr1^−/−^* mice i.t. treated with 2.5 mg/kg poly (I:C) daily for 6 days. (A) Volcano plot of differentially expressed genes (DEGs) between WT and *Ifnlr1^−/−^*. DEGs (*P* < 0.005) with a fold change >1.5 (or <−1.5) are indicated in red; DEGs with a fold change <1.5 (or >−1.5) are in blue. Nonsignificant DEGs (*P* > 0.005) and genes not differentially expressed are indicated in green and gray, respectively. (B and C) Dot plot visualization of gene set enrichment analysis for pathways enriched in (B) WT epithelial cells compared to *Ifnlr1^−/−^* and (C) *Ifnlr1^−/−^* epithelial cells compared to WT. The color of the dots represents the adjusted *P* value (significance) for each enriched pathway; dot size represents the gene set size. FDR, false discovery rate. (**D** and **E**) Epithelial cell proliferation was assessed as 5-ethynyl-2′-deoxyuridine (EdU) incorporation in (D) lung epithelial cells (CD45^−^CD31^−^EPCAM^+^) in WT and *Ifnlr1^−/−^* mice treated as in (A) to (C) or (E) treated as in (A) to (C) and i.t. infected on day 6 with 5 × 10^7^ CFU *S. aureus* for 12 hours. (**F**) Mean fluorescence intensity (MFI) of Ki67 in CD45^−^CD31^−^EPCAM^+^ cells of WT and *Ifnlr1^−/−^* mice treated as in (A) to (C). (**G**) EdU incorporation in lung epithelial cells of WT or *Ifnlr1^−/−^* chimeric mice reconstituted with *Ifnlr1^−/−^* or WT bone marrow treated as in (E). (**H** and **I**) p21 levels in lung epithelial cells (CD45^−^CD31^−^EPCAM^+^) from WT and *Ifnlr1^−/−^* mice treated as in (A) to (C). Representative histogram (H) and MFI (I) are depicted. (A to C) Four mice per genotype. (D and E) Five and (F to I) four mice per group; median and range are represented. (D to I) Representative data of three independent experiments. Statistics: ns, not significant (*P* > 0.05); *P < 0.05; **P < 0.01; ***P < 0.001. (D and E) Two-way ANOVA, (G) one-way ANOVA, and (F and I) and two-tailed *t* tests were performed.

A targeted transcriptomic analysis on lung epithelial cells from mice treated with poly (I:C) revealed a potent down-regulation of the IFN signature in *Ifnlr1^−^*^/^*^−^* compared with WT mice (Fig. 3, A and B, fig. S7, and data S1). This finding confirmed the predominant role of IFN-λ as opposed to type I IFNs during prolonged viral sensing in the lung. Consistent with the observed defect in barrier function, genes associated with apoptosis and the activation of the p53 pathway were enriched in WT compared to *Ifnlr1^−^*^/^*^−^* epithelial cells (Fig. 3B). By contrast, pathways involved in positive regulation of the cell cycle were enriched in *Ifnlr1^−^*^/^*^−^* cells (Fig. 3C). Accordingly, epithelial cells in *Ifnlr1^−^*^/^*^−^* mice, as well as in stromal *Ifnlr1^−^*^/^*^−^* chimeras, proliferated more efficiently after poly (I:C) administration, in the presence or absence of *S. aureus* (Fig. 3, D to G). The most down-regulated gene in *Ifnlr1^−^*^/^*^−^* epithelial cells compared with WT cells was the E3 ubiquitin-protein ligase makorin-1 (*Mkrn1*) (Fig. 3A and data S1). The protein encoded by this gene induces p21 degradation and favors apoptosis via p53 under oxidative stress conditions and after DNA damage (hallmarks of severe viral infections) (*19*). Indeed, *Ifnlr1^−^*^/^*^−^* epithelial cells showed elevated levels of p21 (Fig. 3, H and I). Thus, the ability of IFN-λ to reduce tissue tolerance stems from its capacity to inhibit tissue repair by directly influencing epithelial cell proliferation and viability.

**Fig. 4:**
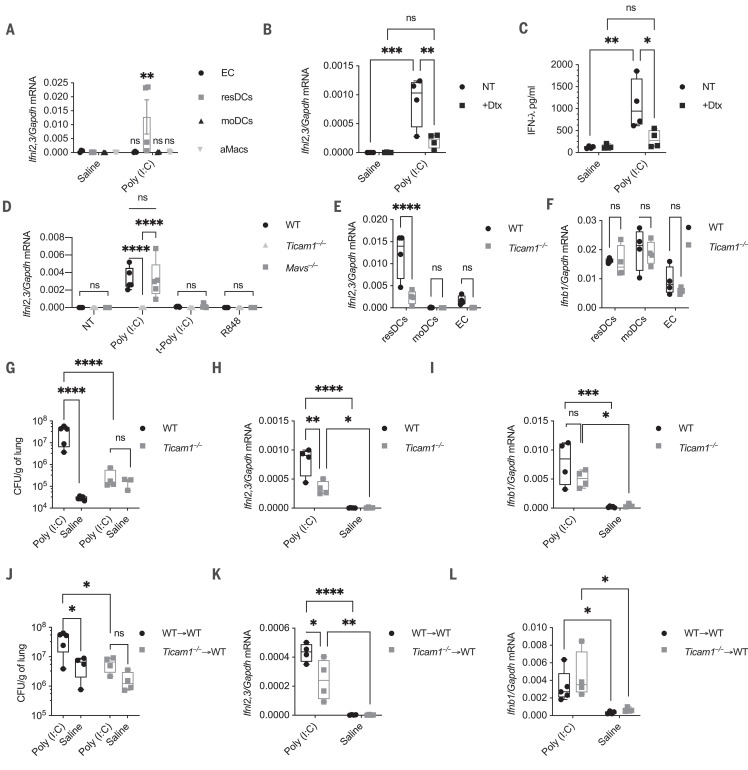
Lung-resident DCs produce IFN-λ downstream of TLR3 upon viral recognition. (**A**) *Ifnl2,3* relative mRNA expression in lung epithelial cells (EC), resident DCs (resDCs), monocyte-derived DCs (moDCs), and alveolar macrophages (aMacs) sorted from WT mice i.t. treated with 2.5 mg/kg poly (I:C) or saline daily for 6 days measured on day 6. (**B** and **C**) CD11c-DTR mice were injected with diphtheria toxin (DTx) to deplete the CD11c^+^ cells in vivo. Relative *Ifnl2,3* mRNA (B) and IFN-λ protein levels (C) from lung homogenates were evaluated on day 6. NT, no toxin. (**D**) DCs differentiated from bone marrow cells in the presence of FMS-like tyrosine kinase 3 ligand (Flt3l) for 9 days from WT, *Ticam1^−/−^*, or *Mavs^−/−^* mice were treated with 50 μg/ml poly (I:C), 1 μg transfected poly (I:C) per 10^6^ cells, or 50 μg/ml R848 for 3 hours. Relative *Ifnl2,3* mRNA expression was evaluated by quantitative polymerase chain reaction. (**E** and **F**) *Ifnl2,3* (E) and *Ifnb1* (F) relative mRNA expression in lung EC, resDCs, and moDCs sorted from WT and *Ticam1^−/−^* mice treated as in (A) was measured on day 6. (**G** to **I**) WT and *Ticam1^−/−^* mice were treated with poly (I:C) as in (A) and subsequently i.t. infected with 5 × 10^7^ CFU of *S. aureus* on day 6 for 12 hours. Lung bacterial burden normalized by lung weight (G), *Ifnl2,3* (H), and *Ifnb1* (I) relative mRNA expression were evaluated. (**J** to **L**) WT chimeric mice reconstituted with *Ticam1^−/−^* bone marrow (*Ticam1^−/−^* → WT) or WT bone marrow (WT → WT) were treated as in (G) to (I). Lung bacterial burden normalized by lung weight (J) and *Ifnl2,3* (K) and *Ifnb1* (L) relative mRNA expression 12 hpi were evaluated. Representative data of three independent experiments are shown. Statistics: ns, not significant (*P* > 0.05); *P < 0.05; **P < 0.01; ***P < 0.001; ***P < 0.001 (two-way ANOVA). Four mice per group; median and range are depicted [(A) to (C), (E) to (L)]. Means ± SEMs of four mice [(A) to (C), (E), and (F)] and of three independent experiments (D) are depicted.

We next investigated the cellular source and molecular pathways that drive IFN-λ production. Upon poly (I:C) administration, lung-resident dendritic cells (DCs) expressed the highest levels of IFN-λ transcript, during both the early and late phases after poly (I:C) administration (Fig. 4A and fig. S8A). By contrast, epithelial cells, alveolar macrophages, and monocytes expressed type I IFNs and proinflammatory cytokines but no IFN-λ transcripts (fig. S8, A to C). Depletion of CD11c^+^ DCs was sufficient to abolish the production of IFN-λ but not type I IFNs (Fig. 4, B and C, and fig. S8, D and E). Alveolar macrophages were not depleted upon diphtheria toxin administration (fig. S8F) and did not produce IFN-λ in response to poly (I:C) (Fig. 4A). By using in vitro–generated DCs, we found that IFN-λ was induced only when the TLR3 pathway was activated (Fig. 4D and fig. S9, A and B). Consistent with in vivo data, TLR7 stimulation in vitro induced only the up-regulation of proinflammatory cytokines (Fig. 4D and fig. S9, A and B). Ex vivo analysis showed that conventional DC1 (cDC1) are the major producer of IFN-λ (fig. S10). Activation of RIG-I and MDA5 via intracellular delivery of poly (I:C) (Fig. 4D and fig. S9, A and B) and of triphosphate hairpin RNA (3p-hpRNA; fig. S11, A to E) induced high levels of type I IFNs, but not type III IFNs, in a mitochondrial antiviral signaling protein (MAVS)–dependent manner. Blockade of endosomal acidification via chloroquine treatment confirmed the importance of TLR3 for IFN-λ induction (fig. S12, A and B). WT mice or mice that do not respond to TLR3 stimulation [Toll-like receptor adaptor molecule 1 deficient (*Ticam1^−^*^/^*^−^*)] were treated in vivo with poly (I:C). Only DCs sorted from *Ticam1^−^*^/^*^−^* mice did not express IFN-λ mRNA, although they still expressed type I IFN mRNA (Fig. 4, E and F). Furthermore, *Ticam1^−^*^/^*^−^* mice were protected against *S. aureus* superinfections (Fig. 4G). *Ticam1^−^*^/^*^−^* mice also showed lower levels of IFN-λ mRNA (but not type I IFN mRNA) than WT mice (Fig. 4, H and I). Similar results were obtained when only hematopoietic cells were deficient in *Ticam1* (Fig. 4, J to L).

The immune system evolved to protect against pathogens, but doing so often threatens host fitness and can cause immunopathologies (*20*). In COVID-19, SARS, MERS, and flu, severe symptoms and death occur late, and after the peak in viral load, indicating a central role for the immune system in driving the pathology (*21*–*24*). In our system, we isolated the effect of immune activation from resistance to lung viral infections and demonstrated that sustained IFN-λ is produced by DCs via TLR3. TLR3 detects replication intermediates from dying cells (*25*) and thus is insensitive to viral immune evasion. Correspondingly, IFN-λ acts on lung epithelial cells and compromises lung barrier function, predisposing the host to lethal secondary bacterial infections.

Previous findings suggested that IFN-λ protects against viral infections (*26*) and increases the barrier functions of gut epithelial cells and endothelial cells (*27*–*29*). These discrepancies may have arisen because, in those studies, the particular cell types targeted by IFN-λ were different. Furthermore, our data support the hypothesis that the detrimental activities of IFN-λ occur only upon chronic exposure and in the presence of tissue damage. Early administration of IFN-λ in a mouse model of COVID-19 could instead confer protection (*30*). Our data enjoin clinicians to carefully analyze the duration of IFN-λ administration and to consider the severity of disease when IFN-λ is used as a therapeutic agent against lung viral infections.

## Supplementary Materials

science.sciencemag.org/content/369/6504/706/suppl/DC1 Materials and Methods Figs. S1 to S13 Tables S1 to S3 MDAR Reproducibility Checklist Data S1

## Appendix

View/request a protocol for this paper from *Bio-protocol*.

## References

[1] A. Broggi, F. Granucci, I. Zanoni, Type III interferons: Balancing tissue tolerance and resistance to pathogen invasion. J. Exp. Med. 217, e20190295 (2020). 10.1084/jem.20190295 31821443 PMC7037241

[2] A. Broggi, Y. Tan, F. Granucci, I. Zanoni, IFN-λ suppresses intestinal inflammation by non-translational regulation of neutrophil function. Nat. Immunol. 18, 1084–1093 (2017). 10.1038/ni.3821 28846084 PMC5701513

[3] I. E. Galani, V. Triantafyllia, E.-E. Eleminiadou, O. Koltsida, A. Stavropoulos, M. Manioudaki, D. Thanos, S. E. Doyle, S. V. Kotenko, K. Thanopoulou, E. Andreakos, Interferon-λ Mediates Non-redundant Front-Line Antiviral Protection against Influenza Virus Infection without Compromising Host Fitness. Immunity 46, 875–890.e6 (2017). 10.1016/j.immuni.2017.04.025 28514692

[4] K. Blazek, H. L. Eames, M. Weiss, A. J. Byrne, D. Perocheau, J. E. Pease, S. Doyle, F. McCann, R. O. Williams, I. A. Udalova, IFN-λ resolves inflammation via suppression of neutrophil infiltration and IL-1β production. J. Exp. Med. 212, 845–853 (2015). 10.1084/jem.20140995 25941255 PMC4451128

[5] M. Merad, J. C. Martin, Pathological inflammation in patients with COVID-19: A key role for monocytes and macrophages. Nat. Rev. Immunol. 20, 355–362 (2020). 10.1038/s41577-020-0331-4 32376901 PMC7201395

[6] L. Prokunina-Olsson, N. Alphonse, R. E. Dickenson, J. E. Durbin, J. S. Glenn, R. Hartmann, S. V. Kotenko, H. M. Lazear, T. R. O’Brien, C. Odendall, O. O. Onabajo, H. Piontkivska, D. M. Santer, N. C. Reich, A. Wack, I. Zanoni, COVID-19 and emerging viral infections: The case for interferon lambda. J. Exp. Med. 217, e20200653 (2020). 10.1084/jem.20200653 32289152 PMC7155807

[7] H. E. Rich, C. C. McCourt, W. Q. Zheng, K. J. McHugh, K. M. Robinson, J. Wang, J. F. Alcorn, Interferon Lambda Inhibits Bacterial Uptake during Influenza Superinfection. Infect. Immun. 87, e00114-19 (2019). 10.1128/IAI.00114-19 30804099 PMC6479047

[8] P. J. Planet, D. Parker, T. S. Cohen, H. Smith, J. D. Leon, C. Ryan, T. J. Hammer, N. Fierer, E. I. Chen, A. S. Prince, Lambda Interferon Restructures the Nasal Microbiome and Increases Susceptibility to Staphylococcus aureus Superinfection. mBio 7, e01939-15 (2016). 10.1128/mBio.01939-15 26861017 PMC4752601

[9] J. A. McCullers, The co-pathogenesis of influenza viruses with bacteria in the lung. Nat. Rev. Microbiol. 12, 252–262 (2014). 10.1038/nrmicro3231 24590244

[10] G. Zhang, C. Hu, L. Luo, F. Fang, Y. Chen, J. Li, Z. Peng, H. Pan, Clinical features and short-term outcomes of 221 patients with COVID-19 in Wuhan, China. J. Clin. Virol. 127, 104364 (2020). 10.1016/j.jcv.2020.104364 32311650 PMC7194884

[11] R. Channappanavar, A. R. Fehr, J. Zheng, C. Wohlford-Lenane, J. E. Abrahante, M. Mack, R. Sompallae, P. B. McCray Jr.., D. K. Meyerholz, S. Perlman, IFN-I response timing relative to virus replication determines MERS coronavirus infection outcomes. J. Clin. Invest. 129, 3625–3639 (2019). 10.1172/JCI126363 31355779 PMC6715373

[12] M. B. Frieman, J. Chen, T. E. Morrison, A. Whitmore, W. Funkhouser, J. M. Ward, E. W. Lamirande, A. Roberts, M. Heise, K. Subbarao, R. S. Baric, SARS-CoV pathogenesis is regulated by a STAT1 dependent but a type I, II and III interferon receptor independent mechanism. PLOS Pathog. 6, e1000849 (2010). 10.1371/journal.ppat.1000849 20386712 PMC2851658

[13] J. Major, S. Crotta, M. Llorian, T. M. McCabe, H. H. Gad, S. L. Priestnall, R. Hartmann, A. Wack, Type I and III interferons disrupt lung epithelial repair during recovery from viral infection. Science 369, 712–717 (2020). 10.1126/science.abc2061 32527928 PMC7292500

[14] D. Blanco-Melo, B. E. Nilsson-Payant, W.-C. Liu, S. Uhl, D. Hoagland, R. Møller, T. X. Jordan, K. Oishi, M. Panis, D. Sachs, T. T. Wang, R. E. Schwartz, J. K. Lim, R. A. Albrecht, B. R. tenOever, Imbalanced Host Response to SARS-CoV-2 Drives Development of COVID-19. Cell 181, 1036–1045.e9 (2020). 10.1016/j.cell.2020.04.026 32416070 PMC7227586

[15] Z. Zhou, L. Ren, L. Zhang, J. Zhong, Y. Xiao, Z. Jia, L. Guo, J. Yang, C. Wang, S. Jiang, D. Yang, G. Zhang, H. Li, F. Chen, Y. Xu, M. Chen, Z. Gao, J. Yang, J. Dong, B. Liu, X. Zhang, W. Wang, K. He, Q. Jin, M. Li, J. Wang, Heightened Innate Immune Responses in the Respiratory Tract of COVID-19 Patients. Cell Host Microbe 27, 883–890.e2 (2020). 10.1016/j.chom.2020.04.017 32407669 PMC7196896

[16] A. Iwasaki, P. S. Pillai, Innate immunity to influenza virus infection. Nat. Rev. Immunol. 14, 315–328 (2014). 10.1038/nri3665 24762827 PMC4104278

[17] H. Kato, O. Takeuchi, S. Sato, M. Yoneyama, M. Yamamoto, K. Matsui, S. Uematsu, A. Jung, T. Kawai, K. J. Ishii, O. Yamaguchi, K. Otsu, T. Tsujimura, C.-S. Koh, C. Reis e Sousa, Y. Matsuura, T. Fujita, S. Akira, Differential roles of MDA5 and RIG-I helicases in the recognition of RNA viruses. Nature 441, 101–105 (2006). 10.1038/nature04734 16625202

[18] A. M. Jamieson, L. Pasman, S. Yu, P. Gamradt, R. J. Homer, T. Decker, R. Medzhitov, Role of tissue protection in lethal respiratory viral-bacterial coinfection. Science 340, 1230–1234 (2013). 10.1126/science.1233632 23618765 PMC3933032

[19] E. W. Lee, M.-S. Lee, S. Camus, J. Ghim, M.-R. Yang, W. Oh, N.-C. Ha, D. P. Lane, J. Song, Differential regulation of p53 and p21 by MKRN1 E3 ligase controls cell cycle arrest and apoptosis. EMBO J. 28, 2100–2113 (2009). 10.1038/emboj.2009.164 19536131 PMC2718286

[20] R. Medzhitov, D. S. Schneider, M. P. Soares, Disease tolerance as a defense strategy. Science 335, 936–941 (2012). 10.1126/science.1214935 22363001 PMC3564547

[21] J. S. Peiris, C. M. Chu, V. C. C. Cheng, K. S. Chan, I. F. N. Hung, L. L. M. Poon, K. I. Law, B. S. F. Tang, T. Y. W. Hon, C. S. Chan, K. H. Chan, J. S. C. Ng, B. J. Zheng, W. L. Ng, R. W. M. Lai, Y. Guan, K. Y. Yuen; HKU/UCH SARS Study Group, Clinical progression and viral load in a community outbreak of coronavirus-associated SARS pneumonia: A prospective study. Lancet 361, 1767–1772 (2003). 10.1016/S0140-6736(03)13412-5 12781535 PMC7112410

[22] Z. A. Memish, S. Perlman, M. D. Van Kerkhove, A. Zumla, Middle East respiratory syndrome. Lancet 395, 1063–1077 (2020). 10.1016/S0140-6736(19)33221-0 32145185 PMC7155742

[23] R. Wölfel, V. M. Corman, W. Guggemos, M. Seilmaier, S. Zange, M. A. Müller, D. Niemeyer, T. C. Jones, P. Vollmar, C. Rothe, M. Hoelscher, T. Bleicker, S. Brünink, J. Schneider, R. Ehmann, K. Zwirglmaier, C. Drosten, C. Wendtner, Virological assessment of hospitalized patients with COVID-2019. Nature 581, 465–469 (2020). 10.1038/s41586-020-2196-x 32235945

[24] C. K. Lee, H. K. Lee, T. P. Loh, F. Y. L. Lai, P. A. Tambyah, L. Chiu, E. S. C. Koay, J. W. Tang, Comparison of pandemic (H1N1) 2009 and seasonal influenza viral loads, Singapore. Emerg. Infect. Dis. 17, 287–291 (2011). 10.3201/eid1702.100282 21291608 PMC3204747

[25] O. Schulz, S. S. Diebold, M. Chen, T. I. Näslund, M. A. Nolte, L. Alexopoulou, Y.-T. Azuma, R. A. Flavell, P. Liljeström, C. Reis e Sousa, Toll-like receptor 3 promotes cross-priming to virus-infected cells. Nature 433, 887–892 (2005). 10.1038/nature03326 15711573

[26] L. Ye, D. Schnepf, P. Staeheli, Interferon-λ orchestrates innate and adaptive mucosal immune responses. Nat. Rev. Immunol. 19, 614–625 (2019). 10.1038/s41577-019-0182-z 31201377

[27] C. Odendall, A. A. Voak, J. C. Kagan, Type III IFNs Are Commonly Induced by Bacteria-Sensing TLRs and Reinforce Epithelial Barriers during Infection. J. Immunol. 199, 3270–3279 (2017). 10.4049/jimmunol.1700250 28954888 PMC5679450

[28] H. M. Lazear, B. P. Daniels, A. K. Pinto, A. C. Huang, S. C. Vick, S. E. Doyle, M. Gale Jr.., R. S. Klein, M. S. Diamond, Interferon-λ restricts West Nile virus neuroinvasion by tightening the blood-brain barrier. Sci. Transl. Med. 7, 284ra59 (2015). 10.1126/scitranslmed.aaa4304 25904743 PMC4435724

[29] F. Douam, Y. E. Soto Albrecht, G. Hrebikova, E. Sadimin, C. Davidson, S. V. Kotenko, A. Ploss, Type III Interferon-Mediated Signaling Is Critical for Controlling Live Attenuated Yellow Fever Virus Infection In Vivo. mBio 8, e00819-17 (2017). 10.1128/mBio.00819-17 28811340 PMC5559630

[30] K. H. Dinnon III, S. R. Leist, A. Schäfer, C. E. Edwards, D. R. Martinez, S. A. Montgomery, A. West, B. L. Yount Jr., Y. J. Hou, L. E. Adams, K. L. Gully, A. J. Brown, E. Huang, M. D. Bryant, I. C. Choong, J. S. Glenn, L. E. Gralinski, T. P. Sheahan, R. S. Baric, A mouse-adapted SARS-CoV-2 model for the evaluation of COVID-19 medical countermeasures. bioRxiv 081497 [Preprint]. 7 May 2020). 10.1101/2020.05.06.081497. 10.1101/2020.05.06.081497

